# Silage fermentation and ruminal degradation of cassava foliage prepared with microbial additive

**DOI:** 10.1186/s13568-019-0906-2

**Published:** 2019-11-09

**Authors:** Mao Li, Xuejuan Zi, Hanlin Zhou, Renlong Lv, Jun Tang, Yimin Cai

**Affiliations:** 1 Tropical Crops Genetic Resources Institute, Chinese Academy of Tropical Agricultural Sciences, Danzhou, 571737 Hainan China; 2 College of Forestry, Hainan University, Danzhou, 571737 Hainan China; 3 Japan International Research Center for Agricultural Sciences, Tsukuba, Ibaraki 305-8686 Japan

**Keywords:** Cassava foliage, Lactic acid bacteria, Cellulase, Silage fermentation, Ruminal degradation

## Abstract

To effectively utilize the tropical cassava (*Manihot esculenta* Crantz) foliage (CF) resources, the CF silages were prepared with microbial additives, including Chikuso-1 (CH1, *Lactobacillus plantarum*), Snow Lact L (SN, *L. rhamnosus*), *Acremonium* cellulase (CE), SN + CE and CH1 + CE. Silage fermentation, chemical composition and ruminal degradation were studied in Hainan, China. CF silages prepared with lactic acid bacteria (LAB) and CE were well preserved, with a higher (*P *< 0.05) lactic acid, a lower (*P *< 0.05) pH value, butyric acid content and NH3-N ⁄ total-N compared with the controls. The additive-treated silages showed increased crude protein (CP) content, but decreased (*P *< 0.05) NDF and ADF contents. Meanwhile, the additive treatment improved relative feed value and ruminal degradability of dry matter (DM), CP, neutral detergent fiber and acid detergent fiber. In addition, the combination of LAB and CE resulted in better fermentation quality and ruminal degradability compared with LAB or CE single treatment. The results demonstrated that the CF could be prepared as ruminant feed, and the combination of LAB and CE might exert beneficial synergistic effect on silage fermentation.

## Introduction

In order to meet the dramatically increased consumption of animal products, the lack of adequate and high-quality green roughage for animal feed has become increasingly prominent with the rapid development of ruminant livestock production based on grassland in China. As an *Euphorbiaceae* woody shrub and major food, bioenergy and feed crop, cassava, *Manihot esculenta* Crantz, is grown in tropics worldwide (Wang et al. 2014). Cassava foliage (CF) can be used as animal feed due to its high contents of protein, gross energy and mineral elements (Li et al. 2019a). Many studies have found that CF can positively affect the digestion, growth performance, carcass characteristics, digestive organ development and gut microbiome diversity of swine, ruminants and poultry (Borin et al. 2006; Oni et al. 2010; Fasae et al. 2011; Nguyen et al. 2012; Régnier et al. 2013; Li et al. 2017, 2019b). Cassava vigorously grows in the summer during rainy seasons, while it stops growing or dies in the cold seasons, leading to feed shortage. CF is normally ensiled after harvest in summer at vegetative stage to ensure continuous supply for ruminants in winter. However, the fermentation quality of CF silage remains low when no additive is applied (Man and Wiktorsson 2002; Napasirth et al. 2015; Li et al. 2019a).

CF is hard to convert to good-quality silage as it often contains low concentrations of water-soluble carbohydrates (WSC) (Napasirth et al. 2015). The quality of silage remains poor when CF is ensiled under natural conditions. Therefore, it is necessary to develop new technologies in order to prepare CF silage with good quality. The commercially available microbial additives, such as lactic acid bacteria (LAB) inoculants, have been developed and widely used for silage preparation (Cai et al. 1999; Napasirth et al. 2015; Guo et al. 2018; Ni et al. 2017; Li et al. 2017, 2019c; Wang et al. 2019; Yang et al. 2019). LAB depletes WSC and creates lactic acid in an anaerobic environment, thus leading to reduced pH and shortened time to reach pH stability. Cellulase enzyme (CE) promotes fiber degradation, elevating the WSC production for LAB to produce lactic acid (Yu et al. 2011; Li et al. 2014, 2017; He et al. 2018). Therefore, LAB and CE can determine the direction of silage fermentation. To the best of our knowledge, limited information is available on the CF silage processed by the commercial LAB inoculant or CE, and their true functions in silage production remain unknown under tropical conditions. In the present study, we aimed to investigate the effects of LAB, CE and their combination on the fermentation quality, chemical composition and ruminal degradation of CF silage.

## Materials and methods

### Silage preparation

The cassava was cultivated at the experimental base of Chinese Academy of Tropical Agricultural Sciences (CATAS), in Danzhou, P. R. China. The CF of approximately 1.5 m plant high was collected and cut into small segments (about 2–3 cm). The CF was wilted for 4 h in the shade. Following treatments were carried out in the present study: control (no additives), LAB inoculant Snow Lact L (SN, *L. Rhamnosus*; Snow Brand Seed Co., Ltd., Sapporo, Japan), Chikuso-1 (CH1, *L. plantarum*; Snow Brand Seed Co., Ltd., Sapporo, Japan), CE (*Acremonium* cellulase, Meiji Seika Pharma Co., Ltd., Tokyo, Japan), SN + CE and CH1 + CE. Each treatment was performed with three replicates. Table 1 lists the production strain, main composition and carboxymethyl cellulase activity of CE used in this study. The application rate of LAB was 1.0 × 10^5^ colony-forming units (cfu)/g of fresh matter (FM), and that of CE was 20 mg/kg of FM. Briefly, 200 g of CF was mixed with additives and kept in plastic bag (30 cm × 10 cm × 4 cm; Menghua Packing Co., Ltd., Guangzhou, China). Properly sealed bags (Jiaren Vacuum Sealer; Jiaren Home Electrical Appliance Co., Ltd., Wuhan, China) were maintained at room temperature (25 to 30 °C). After 30 days of ensiling, chemical composition and fermentation quality were analyzed.

**Table 1:** CMCase activity of CE used in this study

	Cellulase
Production strain	*Acremonium cellulolyticus*
Main composition	Glucanase, pectinase
CMCase activity	7350 U/g

*CMCase* carboxymethyl-cellulase

### Chemical analysis

Samples were dried at 65 °C for 48 h and milled through a 1.0-mm sieve for chemical analysis. Dry matter (DM), crude protein (CP), organic matter (OM) and ether extracts (EE) were determined based on previously established methods (AOAC 1990). Neutral detergent fiber (NDF) and acid detergent fiber (ADF) were determined by the methods of Van Soest et al. (1991). Heat-stable amylase and sodium sulfite were used during NDF procedure. Relative feed value (RFV) of the CF samples was calculated as previously described (Rohweder et al. 1978). Table 2 shows the chemical compositions of fresh CF. The RFV was calculated according to the equation:

$${\text{RFV}}\;\left( \% \right) = \frac{{\left[ { 8 8. 9 { } - \, \left( { 0. 7 7 9 { } \times {\text{ADF}}} \right)} \right] \, \times \, \left( { 1 2 0\div {\text{NDF}}} \right)}}{1.29}$$

**Table 2:** Chemical composition of CF

	DM (%)	OM (% DM)	EE (% DM)	CP (% DM)	WSC (% DM)	NDF (% DM)	ADF (% DM)	RFV
Cassava foliage	24.80	92.00	5.73	22.67	8.21	41.19	33.88	141.17

*DM* dry matter, *OM* organic matter, *EE* ether extract, *CP* crude protein, *WSC* water-soluble carbohydrates, *NDF* neutral detergent fiber, *ADF* acid detergent fiber, *RFV* relative feed value

The fermentation products of silages were analyzed using cold-water extracts. Briefly, 50 g wet silage was mixed with 200 mL distilled water and incubated at 4 °C overnight. The pH, organic acids (lactic acid, acetic acid, propionic acid and butyric acid) and NH_3_-N were determined by the methods of Li et al. (2017).

### Ruminal degradability analysis

The animal-related protocols were approved by the Animal Care and Use Committee of CATAS, P. R. China, and trials were carried out at CATAS in August 2017. Three healthy mature Hainan black goats were ruminally cannulated to compare the in situ ruminal degradability of CF silages. The CF silages ruminal degradability of DM, CP, NDF and ADF were determined as previously described by Li et al. (2017).

### Statistical analysis

A completely randomized design was applied to the data of silages, which were analyzed using the general linear models (GLM) of SAS (1996). Differences among various treatments were analyzed using probability of difference. Duncan’s multiple range tests were employed to compare significant differences, and *P *< 0.05 was considered as statistically significant.

## Results

### Chemical composition of fresh CF and silages

Table 3 lists the chemical compositions of CF silages. Compared with the control, the additive treatments increased (*P *< 0.05) DM contents of CF silage, while there were no great differences among additive-treated silages. The OM and EE contents were similar in all treatments. The CP contents of CH1 and CE-treated CF silages were higher compared with the control group, and in combination treatments (CH1 + CE or SN + CE) were higher (*P *< 0.05) than those of single treatment and control. In contrast, the CE and combination treatments decreased (*P *< 0.05) the ADF and NDF contents of CF silage compared with the control group. In addition, the ADF and NDF contents of CF silage treated with combination of LAB and CE were decreased (*P *< 0.05) compared with the CF silage treated with single additives. The additive treatments increased (*P *< 0.05) the RFV, and the RFV in the combination treatments (CH1 + CE or SN + CE) was higher (*P *< 0.05) than single additive treatments (*P *< 0.05). Compared with the CH1 and SN treatments, the CE treatment had a higher RFV (*P *< 0.05).

**Table 3:** Chemical composition of CF silage

Treatments	DM (%)	OM (% DM)	EE (% DM)	CP (% DM)	ADF (% DM)	NDF (% DM)	RFV
Control	32.52b	91.39	6.88	21.55b	30.48a	41.39a	146.45c
SN	34.21a	90.52	6.62	21.09b	30.49a	40.72a	148.84c
CE	34.36a	89.77	6.57	22.26b	26.01b	38.83b	164.42b
CH1	34.51a	89.62	6.83	21.85b	30.17a	40.56a	149.99c
SN + CE	34.75a	90.14	6.77	24.84a	22.59c	33.96c	195.31a
CH1 + CE	34.60a	92.10	6.92	24.20a	21.67c	35.26c	190.01a
SEM	0.45	0.39	0.06	0.62	1.66	1.27	8.89
*P* value	0.031	0.789	0.342	0.024	0.008	0.009	0.003

CH1: *L. plantarum*; SN: *L. Rhamnosus*; CE: cellulase enzyme; SN + CE: *L. plantarum* + cellulase enzyme; CH1 + CE: *L. Rhamnosus* + cellulase enzyme

*DM* dry matter, *OM* organic matter, *EE* ether extract, *CP* crude protein, *NDF* neutral detergent fiber, *ADF* acid detergent fiber, *RFV* relative feed value, *SEM* standard error of means

Means within the same column with different letters are significantly different (P < 0.05)

### Fermentation quality of CF silages

Table 4 shows the fermentation quality of CF silages. Compared with the control group, the additive treatments decreased pH of CF silage (*P *< 0.05), and the pH of the combination treatments was lower (*P *< 0.05) than the single additive treatments. The LAB treatments increased (*P *< 0.05) the lactic acid content compared with the control group, and the lactic acid content in CE, CH1 + CE and SN + CE groups was higher (*P *< 0.05) compared the LAB treatment. The acetic acid content and NH_3_-N ⁄ total-N in additive treatments were lower compared with the control group, and they were lower (*P *< 0.05) in CH1 + CE and SN + CE groups than compared with the LAB treatment. The propionic acid content remained relatively stable in all treatments. The butyric acid content in additive treatments was lower (*P *< 0.05) compared with the control group, and such acid content in CH1 + CE and SN + CE groups was lower (*P *< 0.05) compared with the other treatments.

**Table 4:** Fermentation quality of CF silage

Treatments	pH	Lactic acid (% DM)	Acetic acid (% DM)	Propionic acid (% DM)	Butyric acid (% DM)	NH3-N ⁄Total-N (% DM)
Control	4.73a	0.22c	1.68a	0.79a	0.23a	2.16a
SN	4.43b	1.08b	1.38b	0.64a	0.17b	1.84b
CE	4.33b	3.01a	1.20c	0.63a	0.21ab	1.25bc
CH1	4.38b	1.10b	1.41b	0.72a	0.19b	1.48b
SN + CE	4.09c	3.26a	1.25c	0.71a	0.09c	1.31bc
CH1 + CE	4.11c	3.46a	0.72d	0.69a	0.07c	1.16c
SEM	0.09	0.564	0.13	0.02	0.03	0.16
P-value	0.005	0.003	0.009	0.054	0.023	0.018

CH1, *L. plantarum*; SN, *L. Rhamnosus*; CE, cellulase enzyme; SN + CE, *L. plantarum* + cellulase enzyme; CH1 + CE, *L. Rhamnosus* + cellulase enzyme

*DM* dry matter, *SEM* standard error of means

Means within the same column with different letters are significantly different (P < 0.05)

### Ruminal degradability of CF silages

Table 5 shows the ruminal degradability of CF silage. The DM, CP, ADF and NDF degradability of CF silage treated with LAB and CE were higher compared with the control group, and these values were higher (*P *< 0.05) in CH1 + CE and SN + CE groups compared with the LAB or CE treatment.

**Table 5:** Ruminal degradability of CF silage

Treatments	DM degradability (%)	CP degradability (%)	NDF degradability (%)	ADF degradability (%)
Control	83.43b	70.47c	70.28b	72.35b
SN	84.52b	75.09b	71.45b	72.63b
CE	84.81b	76.83b	72.61b	73.50b
CH1	85.22b	75.08b	70.84b	72.33b
SN + CE	89.75a	79.46a	77.84a	75.20a
CH1 + CE	89.06a	79.12a	78.38a	75.44a
SEM	0.56	1.27	1.34	0.58
P-value	0.016	0.012	0.021	0.034

CH1, *L. plantarum*; SN, *L. Rhamnosus*; CE, cellulase enzyme; SN + CE, *L. plantarum* + cellulase enzyme; CH1 + CE, *L. Rhamnosus* + cellulase enzyme

*DM* dry matter, *CP* crude protein, *NDF* neutral detergent fiber, *ADF* acid detergent fiber, *SEM* standard error of means

Means within the same column with different letters are significantly different (P < 0.05)

## Discussion

### Chemical composition

Generally speaking, CF has relatively low WSC content and less epiphytic LAB, leading to poor fermentation quality of silages without additives (Napasirth et al. 2015; Li et al. 2019a). It is necessary to use microbial inoculants to control silage fermentation during ensiling (Cai et al. 1999; Li et al. 2017; Wang et al. 2019). Moisture of material is also an important factor affecting silage fermentation. In the present study, additive treatments increased the DM of CF silage, which is consistent with previous studies on other silages (Kung and Ranjit 2001; Li et al. 2014, 2017; Wang et al. 2019). This could be attributed to that the additives treatment promoted the growth and propagation of LAB, which could inhibit the growth of aerobic and anaerobic bacteria by the lower pH, then reduce nutrient consumption of these microbial keep more nutrient substance and result in higher DM. Such elevation in CP content could be attributed to the concentration effect due to the loss of organic carbon during fermentation or the combination of proteolysis inhibition and concentration effect (He et al. 2018). However, the mechanism underlying such finding remains largely unexplored. We found that the CF silage treated with LAB or CE had higher RFV and lower NDF and ADF contents compared with the control treatment. Consistently, few studies have reported that CE can decrease the fiber fractions (NDF and ADF) of silages (Liu et al. 2012; Li et al. 2014, 2017; Chen et al. 2016; Ni et al. 2017). These results could be explained by that CE increased the availability of WSC derived from fiber by enzymolysis and acid solubilization, leading to increased availability of fermentation substrates for LAB. Moreover, CE promote fermentation and fiber degradation. Taken together, LAB and CE treatments resulted in less degradation of protein and more degradation of fiber during ensiling, by which more nutrients were preserved in CF silage.

### Fermentation quality

The fermentation quality of silage is the result of the combined effects of pH, lactic acid, volatile acid composition and NH_3_-N⁄total-N as well as other factors. LAB should be dominant in the fermentation process of the good silage, which can accelerate the fermentation process and improve the fermentation quality (Cai et al. 1999). In this study, the LAB or CE treatment reduced the pH and NH_3_-N content, elevated the content of lactic acid, and ameliorated the fermentation quality of silage compared with the control treatment. Similar effects on other silage fermentation have been achieved by applying LAB and CE (Colombatto et al. 2003; Kung et al. 2003; Napasirth et al. 2015; Chen et al. 2016; Ni et al. 2017; Li et al. 2017, 2019c). We also found that the CE treatment led to a higher production of lactic acid and a lower concentration of acetic acid. This finding suggested that the LAB used in this study was driven toward homo-fermentation type of lactic acid, resulting in promoted silage fermentation. Furthermore, the combination of LAB and CE was more effective compared with LAB or CE single treatment, indicating that there was a synergistic effect on silage. Perhaps the cellulase hydrolysis of fiber fractions increased the availability of WSC acting as a fermentation substrate of LAB and produced more lactic acid, leading to reduced pH and improved fermentation quality (Ni et al. 2017; Li et al. 2017, 2019c).

### Ruminal degradability

Digestibility of forage is one of the most important evaluation indexes of feeding value, which affects feed intake and greatly relies on its chemical compositions, especially the fiber fraction and structure (Chabot et al. 2008). Previous studies have reported that LAB or CE additives have either positive effect (Cai et al. 2003; Cao et al. 2010; Li et al. 2014; Moselhy et al. 2015; Bureenok et al. 2016; Chen et al. 2016; Li et al. 2017; He et al. 2018) or no effect (Jaakkola et al. 1991; Zahiroddini et al. 2004; Moharrery et al. 2009; Fang et al. 2012; Ellis et al. 2016) on degradability improvement. In our study, LAB or CE treatment enhanced the ruminal degradability of CF silage. The possibility mechanism could be that the addition of LAB and enzymes destroyed the structure of plant cell wall, effectively released the intracellular contents, supplied more fermentation substrate for rumen microorganisms, and then improved the ruminal degradability (Yu et al. 2011; Li et al. 2014; Chen et al. 2016; Li et al. 2017). The ruminal degradability of different forages could be differently impaired by LAB or CE. However, such discrepancy may be attributed to characteristic differences in forage materials, especially their chemical compositions. Furthermore, the higher degradability of the CF silages can be attributed to their high CP content and low fiber, which provide more fermentation substrate for rumen microorganisms, then promoted rumen digestion. Besides, the appropriate carbon–nitrogen ratio or the protein structure of CF silage is easily digestible. Low ruminal degradability has been reported in both typical tropical forages, King grass with low CP and high fiber and Stylo with moderate CP and high fiber, in neither of which the carbon–nitrogen ratio is appropriate (Li et al. 2014, 2017; Zhang et al. 2018). Therefore, a reasonable combination of CF and other tropical forages could maximize the use of local feed resources, promote the balance of animal diets and improve animal performance.

The fermentation quality, chemical composition and ruminal degradability of CF silage prepared with commercial LAB inoculant and CE in tropics were studied. The LAB and CE could effectively improve the fermentation quality, chemical composition and ruminal degradability compared with the control group, and the combination of LAB and CE displayed more effective results. The results confirmed that the CF could be prepared into good-quality silage, and the combination of LAB and CE had a beneficial synergistic effect on silage fermentation.

## Data Availability

Not applicable.

## Abbreviations

CF: cassava foliage
CE: cellulase enzyme
LAB: lactic acid bacteria
CP: crude protein
DM: dry matter
WSC: water-soluble carbohydrates
CATAS: Chinese Academy of Tropical Agricultural Sciences
FM: fresh matter
OM: organic matter
EE: ether extracts
NDF: neutral detergent fiber
ADF: acid detergent fiber
RFV: relative feed value
GLM: general linear models
